# COLLABORATIVE NEEDS ASSESSMENT OF RURAL OLDER ADULTS AND PRIORITIES FOR AGING IN PLACE

**DOI:** 10.1093/geroni/igac059.889

**Published:** 2022-12-20

**Authors:** Bernard Steinman, Jennifer Tabler, Jeff Clark, Lisa Osvold

**Affiliations:** University of Wyoming, Laramie, Wyoming, United States; University of Wyoming, Laramie, Wyoming, United States; Wyoming Department of Health Aging Division, Cheyenne, Wyoming, United States; Wyoming Department of Health - Aging Division, Cheyenne, Wyoming, United States

## Abstract

To assess aging-related needs of state residents, the Wyoming GWEP collaborated with the state’s Department of Health--Aging Division to conduct a state-wide survey as the basis for Wyoming’s 4-year state plan on aging. Survey items addressed concerns related to community living and caregiving, as well as general concerns related to aging in Wyoming’s rural setting. Results suggest older residents are committed to aging at home; and when that is not feasible, wish to remain in their communities when possible. Concerns were identified regarding attributes of the state that place older residents at risk for poorer physical/mental health, dependence, financial jeopardy, social isolation, and negative secondary outcomes that are comparatively more costly to address in institutional settings. We discuss the collaborative needs-assessment process, as well as priorities for future service delivery that promote good social support, transportation options, and home modifications to increase independence and safety.

